# Isolation of Adipose-Derived Stem/Stromal Cells from Cryopreserved Fat Tissue and Transplantation into Rats with Spinal Cord Injury

**DOI:** 10.3390/ijms19071963

**Published:** 2018-07-05

**Authors:** Yuki Ohta, Mitsuko Takenaga, Akemi Hamaguchi, Masanori Ootaki, Yuko Takeba, Tsukasa Kobayashi, Minoru Watanabe, Taroh Iiri, Naoki Matsumoto

**Affiliations:** 1 Department of Pharmacology, St. Marianna University School of Medicine, 2-16-1 Sugao, Miyamae-ku, Kawasaki, Kanagawa 216-8511, Japan; m_ootaki@marianna-u.ac.jp (M.O.); takebay@marianna-u.ac.jp (Y.T.); kobayashit@marianna-u.ac.jp (T.K.); tiiri@marianna-u.ac.jp (T.I.); matsumoto@marianna-u.ac.jp (N.M.); 2 Institute of Medical Science, St. Marianna University School of Medicine, 2-16-1 Sugao, Miyamae-ku, Kawasaki, Kanagawa 216-8512, Japan; m2take@marianna-u.ac.jp (M.T.); hamaguch@marianna-u.ac.jp (A.H.); 3 Institute for Animal Experimentation, St. Marianna University Graduate School of Medicine, 2-16-1 Sugao, Miyamae-ku, Kawasaki, Kanagawa 216-8511, Japan; m4wata@marianna-u.ac.jp

**Keywords:** adipose tissue, adipose-derived stem/stromal cells, cryopreservation, spinal cord injury, transplantation, tissue bank

## Abstract

Adipose tissue contains multipotent cells known as adipose-derived stem/stromal cells (ASCs), which have therapeutic potential for various diseases. Although the demand for adipose tissue for research use remains high, no adipose tissue bank exists. In this study, we attempted to isolate ASCs from cryopreserved adipose tissue with the aim of developing a banking system. ASCs were isolated from fresh and cryopreserved adipose tissue of rats and compared for proliferation (doubling time), differentiation capability (adipocytes), and cytokine (hepatocyte growth factor and vascular endothelial growth factor) secretion. Finally, ASCs (2.5 × 10^6^) were intravenously infused into rats with spinal cord injury, after which hindlimb motor function was evaluated. Isolation and culture of ASCs from cryopreserved adipose tissue were possible, and their characteristics were not significantly different from those of fresh tissue. Transplantation of ASCs derived from cryopreserved tissue significantly promoted restoration of hindlimb movement function in injured model rats. These results indicate that cryopreservation of adipose tissue may be an option for clinical application.

## 1. Introduction

Regenerative medicine has advanced rapidly in recent years. Embryonic stem (ES) cells, induced pluripotent stem (iPS) cells, and somatic stem cells have all become candidates for transplantation in regenerative therapy. However, ES or iPS cells have some limitations to their clinical application, including ethical concerns, problems with immunogenicity, and safety issues related to the potential for tumorigenicity [1]. Somatic stem cells, such as mesenchymal stem cells (MSCs), may offset these problems, as they are capable of self-renewal and can differentiate into multi-lineages [2,3,4]. In addition, MSCs are known to have low immunogenicity [5]. Therefore, MSCs are prioritized over ES or iPS cells in clinical trials. MSCs have been found in many tissues, including adipose tissue, which possesses its own advantages for use in regenerative medicine [6,7,8]. These advantages include its ubiquitous distribution, its accessibility, and the ease with which it can be harvested. Moreover, it contains two kinds of regenerative cells, de-differentiated fat cells (DFAT) and adipose-derived stem/stromal cells (ASCs). Spinal cord injury (SCI) results in partial or complete paralysis and disability. Currently, cell transplantation is being explored as a strategy to promote tissue regeneration, and a variety of cells have been tested for their therapeutic potential for SCI [9,10,11]. We have reported that the transplantation of DFAT or ASCs improved hindlimb motor dysfunction in rats with SCI [12,13]. The mechanism of their treatment in this injury may be paracrine effect by humoral factors, not neuronal differentiation [12,13].

For research use, the demand for adipose tissue remains high, but its supply system is lacking. We have operated the human tissue bank since 2001, gathering liver and small intestine tissue that have been removed by surgery and have been judged unnecessary for diagnosis. Although we began gathering adipose tissue, whether or not cells for transplantation can be obtained from cryopreserved tissue remains unknown. In this study, we attempted to isolate ASCs from cryopreserved adipose tissue, with the aim of evaluating adipose tissue banking as a source of cells for regenerative medicine, including SCI.

## 2. Results

### 2.1. Isolation of ASCs from Cryopreserved Adipose Tissue

**Figure 1:**
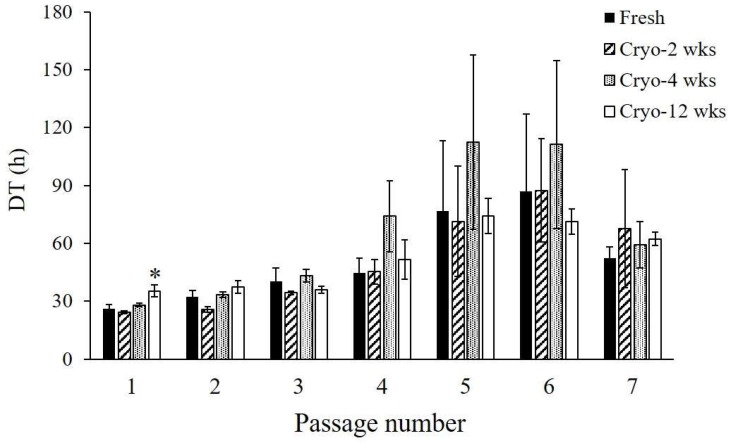
Doubling time of adipose-derived stem/stromal cells (ASCs). ASCs derived from fresh or cryopreserved adipose tissue were cultured for up to seven passages. Doubling time (DT) was determined at the indicated number of passages (as described in the Materials and Methods section). Data are expressed as the mean ± standard error (SE) (*n =* 4). * *p* < 0.05 vs. Fresh.

Adherent cells were observed the day after seeding and isolated from fresh adipose tissue with collagenase. ASCs became apparently homogeneous during culture and passage, although the isolated cells exhibited heterogeneous features. When cryopreserved tissues were treated with collagenase, the cells adhered on the culture dish and grew, similar to the treatment of fresh tissue. ASCs isolated from two- and four-week cryopreserved tissue (Cryo-ASCs) could be amplified with a doubling time (DT) of approximately 24–28 h at the first passage (P1), while ASCs isolated from fresh tissue (Fresh-ASCs) showed a doubling time of approximately 26 h (Figure 1). DT was longer in the 12-week-Cryo-ASCs (approximately 35 h) than in Fresh-ASCs (*n* = 4, *p* < 0.05). Culture and passage progressively lengthened DT in all Fresh- and Cryo-ASCs, with P5 or P6 as the peak (Figure 1). There was no significant difference in the proliferative activity between the two.

### 2.2. Adipogenic and Osteogenic Differentiation

**Figure 2:**
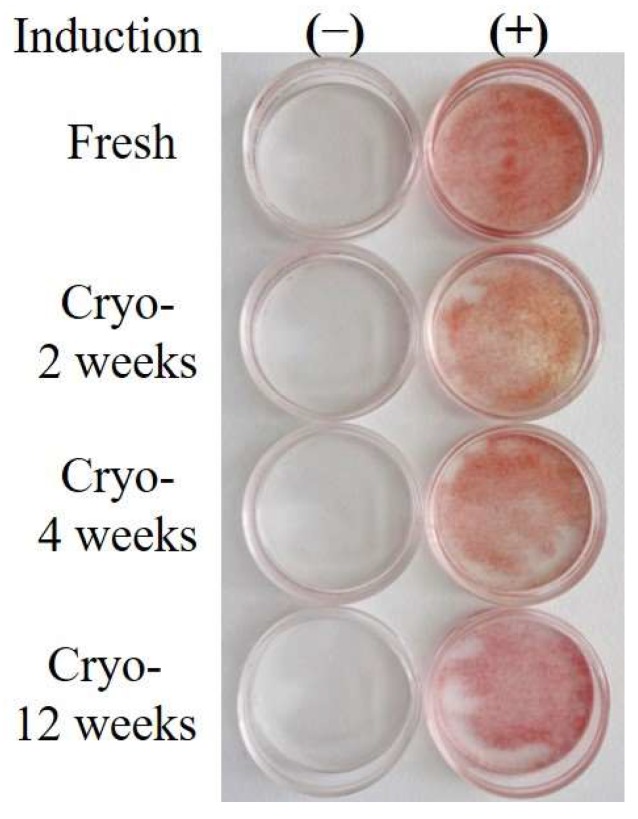
Adipogenic differentiation in ASCs. Fresh or cryopreserved adipose-derived stem/stromal cells (Fresh- or Cryo-ASCs) were differentiated into adipocytes (as described in the Materials and Methods section). Adipogenesis was revealed by Oil red O staining for lipid droplets. (−): confluent state without adipogenic induction; (+): 28 days after adipogenic induction.

Adipogenic differentiation of Fresh- and Cryo-ASCs was evaluated. When confluent cells were subsequently cultured in adipogenic medium, intracellular lipid droplets formed (Figure 2). Adipose differentiation was observed in both Fresh- and Cryo-ASCs. However, the efficiency of adipogenic differentiation was lower in 12-week-Cryo-ASCs (Table 1).

**Figure 3:**
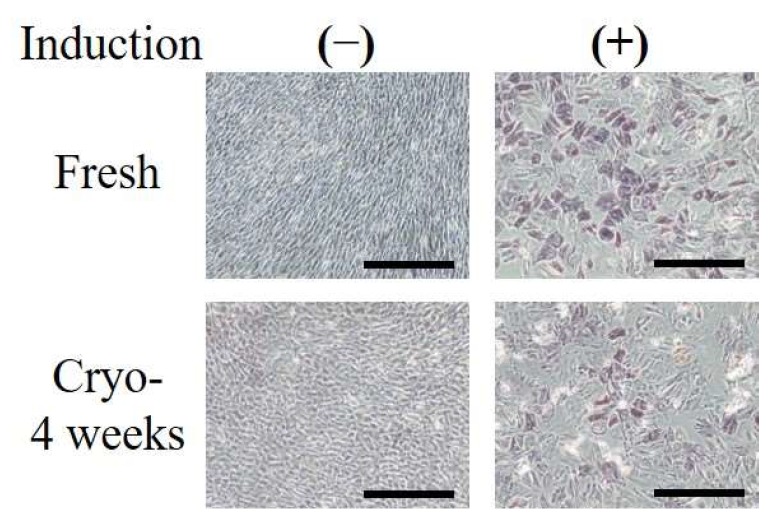
Osteogenic differentiation in ASCs. Fresh or Cryo-ASCs were differentiated into osteoblasts (as described in the Materials and Methods section). Osteogenesis was revealed by alkaline phosphatase staining. (−): confluent state without osteogenic induction; (+): 21 days after osteogenic induction. Scale bars = 500 µm.

When Fresh- or Cryo-ASCs were cultured in osteogenic medium, the positive cells for alkaline phosphatase (ALP) were observed (Figure 3).

### 2.3. Secretion of Cytokines

**Figure 4:**
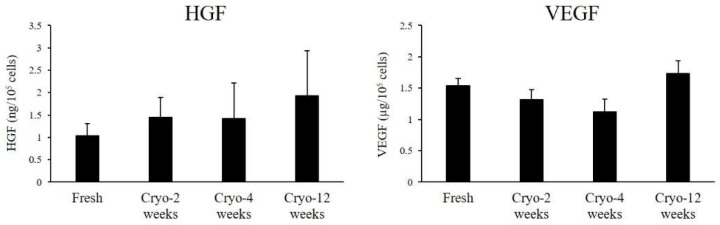
Cytokine secretion from ASCs. P2-ASCs were cultured for four days. Supernatants were harvested for cytokine assay. Hepatocyte growth factor (HGF) and vascular endothelial growth factor (VEGF) in the culture medium were measured by enzyme-linked immunosorbent assay. Data are expressed as the mean ± SE (*n* = 4).

To investigate the effect of cryopreservation on cytokine secretion from ASCs, hepatocyte growth factor (HGF) and vascular endothelial growth factor (VEGF) were measured in their culture media. Both Fresh- and Cryo-ASCs secreted large amounts of HGF and VEGF in vitro (Figure 4).

### 2.4. Transplantation of ASCs into Rats with SCI

**Figure 5:**
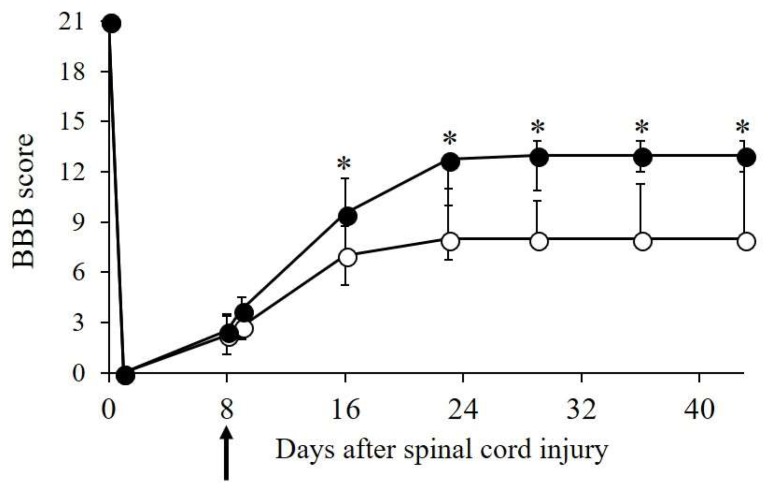
Functional recovery following ASC transplantation. On day eight after injury (arrow), 12-week-Cryo-ASCs (2.5 × 10^6^ cells) were infused into spinal cord injury (SCI) rats via the tail vein (*n* = 8). Basso–Beattie–Bresnahan (BBB) scores were determined before and after transplantation. Black and white circles are ASC and saline group, respectively. The median score is plotted; bars show the inter quartile range (IQR). * *p* < 0.05 vs. saline controls (*n* = 10).

Next, we investigated whether Cryo-ASCs were effective for improvement of SCI. SCI caused complete hindlimb paralysis, after which gradual recovery of locomotion was observed. As shown in Figure 5, the Cryo-ASC group showed greater improvement than the saline group in hindlimb locomotive function during the early period after transplantation. The Basso–Beattie–Bresnahan (BBB) score of all SCI animals reached a plateau at four weeks after injury, though ASC-treated animals showed significantly better recovery (median: 13, interquartile range (IQR): 3) than the saline group (median: 8, IQR: 2.75) (*p* < 0.05). In the ASC group, some animals showed frequent-consistent weight-bearing plantar walking with good forelimb–hindlimb coordination. While the saline group also recovered, some animals crawled and moved without weight-bearing.

### 2.5. Long-Term Cryopreservation of Adipose Tissue

**Figure 6:**
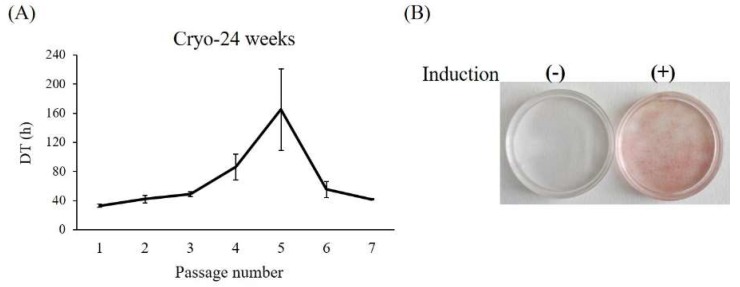
Long-term cryopreserved adipose-tissue-derived cells. ASCs were isolated from 24-week-cryopreserved tissues and examined for proliferation and adipogenic differentiation: (**A**) DT; (**B**) Oil red O staining. Data are expressed as the mean ± SE (*n* = 4).

Finally, we attempted to isolate ASCs from adipose tissue that had undergone long-term cryopreservation (24 weeks). ASCs with proliferative patterns similar to those of Fresh-ASCs were obtained. Moreover, there was no significant difference in proliferative activity between the two, although DT at P1 was longer (approximately 33 h, Figure 6A) in Cryo-ASCs. Adipogenic differentiation was even lower in Cryo-ASCs (Figure 6B), which showed a 25% (1/4) ratio of adipocyte differentiation.

## 3. Discussion

The present study demonstrated that the isolation of ASCs from cryopreserved tissue was possible, and a therapeutic effect was observed when they were transplanted into rats with SCI. In fact, intravenous infusion of Cryo-ASCs into SCI rats promoted the restoration of motor function, just as we observed in our previous study using Fresh-ASCs [13]. Therefore, transplantation of Cryo-ASCs improved motor function in SCI rats just as Fresh-ASCs did.

When comparing Fresh- and Cryo-ASCs, the cell proliferative patterns were similar, while DTs at P1 tended to be longer with a longer cryopreservation period. In addition, cryopreservation has been reported to not alter expression of cell surface markers [14], characterizing ASCs. ASCs are known to secrete angiogenic growth factors such as HGF and VEGF [15]. The paracrine effect of cytokines secreted from ASCs is considered to play a major role in cell therapy, including SCI [16]. In our study, Fresh- and Cryo-ASCs were equivalent in cytokine secretion, with both Fresh- and Cryo-ASCs secreting abundant growth factors. On the other hand, adipogenic differentiation decreased as cryopreservation time increased. These results indicate that cryopreservation might have affected cell differentiation but did not affect cell proliferation and cytokine secretion. For other mesenchymal lineage differentiation abilities, the positive cells were recorded when stained for alkaline phosphatase activity as an evaluation of osteogenic differentiation. However, in addition to osteogenic differentiation, more detailed studies are needed for chondrogenic differentiation ability.

ASCs were successfully isolated from tissues cryopreserved at −80 °C for six months. However, adipose differentiation was unsatisfactory. Therefore, we will need to reconsider the temperature and agent for cryopreservation of adipose tissue. Indeed, even after this study, optimum conditions for the storage of adipose tissue remain unknown, and previous studies have produced a variety of results. For example, some studies reported that storage at −20 °C was comparable to storage in liquid nitrogen (−196 °C), while others reported that storage in liquid nitrogen was favorable to storage at −20 °C; and still others reported that storage at −80 and −196 °C was equivalent [17,18,19,20]. In clinical application, Cryo-ASCs are most likely to be used as allograft rather than autograft. There will be not concerns about the potential risks of immuno-response even in such circumstances because MSCs containing ASCs are known to have low immunogenicity, and immunologically tolerant [5,21].

In conclusion, ASCs were obtained from cryopreserved adipose tissue, and, with the exception of adipose differentiation, they were functionally equivalent to freshly isolated ASCs. These findings show that banking of adipose tissue is possible, suggesting the use of cryopreserved adipose tissue in future clinical applications.

## 4. Materials and Methods

### 4.1. Animals

Female Sprague–Dawley rats (Charles River Laboratories Japan, Yokohama, Japan) were housed in an animal room at a constant temperature (23 ± 1 °C) and humidity (50%–60%) with a 12-h light/dark cycle and were allowed free access to a standard diet and water. Experimental procedures were performed in accordance with the Guidelines for Animal Experimentation of St. Marianna University Graduate School of Medicine (Kawasaki, Japan). The experimental protocol was approved by the Animal Research Committee, Institute for Animal Experimentation, St. Marianna University Graduate School of Medicine (approval number 1310005, 25 October 2013).

### 4.2. Cryopreservation of Adipose Tissue

Dorsal fat pads were obtained from eight–nine-week-old rats (*n* = 4) under anesthesia. The weight of adipose tissues was measured, and they were divided into five parts. Adipose tissues were soaked in a cryoprotectant (TC-Protecter, DS Pharma Biomedical, Osaka, Japan), and then stored in a freezer at −80 °C.

### 4.3. Isolation of ASCs

Frozen tissues were fast-thawed in a water bath at 37 °C and washed with phosphate-buffered saline (PBS). The fresh or thawed adipose tissue was minced and digested for 1 h in 0.1% collagenase (Thermo Fisher Scientific, Waltham, MA, USA) at 37 °C. The digestion process was stopped by adding Dulbecco’s Modified Eagle’s Medium (DMEM, Thermo Fisher Scientific) containing 20% fetal bovine serum (FBS, Thermo Fisher Scientific) and 1% antibiotic-antimycotic (Thermo Fisher Scientific). The digested suspension was filtered through a 70-µm nylon mesh cell strainer (BD Biosciences, Franklin Lakes, NJ, USA) to remove tissue debris and centrifuged at 1000 rpm for 5 min. The pellet (stromal-vascular fraction) was suspended in medium and cultured in a 10-cm culture dish at 37 °C in a humidified atmosphere of 5% CO_2_ and 95% room air. After 24 h, non-adherent cells were gently removed. When adherent cells reached 80% confluence (passage 0: P0), confluent cells (ASCs) were detached with 0.25% trypsin/1 mM ethylenediamine tetraacetic acid (EDTA) (Thermo Fisher Scientific) and plated in flasks at a density of 2.5 × 10^5^ cells/75 cm^2^ (P1). DT was determined by manually counting the number of cells from P1 to P7. The numbers of cells and culture times (CT) were used to calculated DTs based on the following formula: DT = log 2 × CT/(log N − log N_0_) where N_0_ represents the initial cell number and N represents cell number at the time of harvest.

### 4.4. Adipogenic and Osteogenic Differentiation

Differentiation into adipocytes was performed using a modification of the previously described methods [2,22]. Confluent cells were incubated in adipogenic induction medium (a 3:1 mixture of DMEM and Ham’s F-12 Medium (Thermo Fisher Scientific) containing 10% FBS, 1% antibiotic-antimycotic, 5 µg/mL insulin (Thermo Fisher Scientific), 5 µg/mL transferrin (Thermo Fisher Scientific), 0.25 µM dexamethasone (DEX, MP Biomedicals, Aurora, OH, USA), and 500 µM 3-isobutyl-1-methylxanthine (IBMX, Wako, Osaka, Japan)) for three days, followed by culture in maintenance medium (induction medium without DEX and IBMX) for several days. After this culture procedure was repeated three times, the cells were cultured in maintenance medium for seven days. Cells were fixed in 10% phosphate-buffered formalin for 1 h and stained with Oil Red O (Sigma-Aldrich, St. Louis, MO, USA) to visualize lipid droplet accumulation.

Differentiation into osteoblasts was performed using Osteoblast-Inducer Reagent (Takara Bio, Shiga, Japan). ASCs were incubated in osteoblastic induction medium (DMEM containing 20% FBS, 5 µg/ml ascorbic acid, 100 nM hydrocortisone, and 10 mM β-glycerophosphate) for 21 days. The medium change was performed each three day. ALP as an osteoblast marker was detected by an ALP stain kit according to the manufacturer’s instructions (Takara Bio).

### 4.5. Measurement of Cytokines

After P2-ASCs were cultured for four days, the supernatant was collected for the measurement of cytokines, and the number of cells was counted with a hemocytometer. After centrifuged at 1000 rpm for 5 min, and aliquots of supernatant were used for assay.

Levels of HGF (Institute of Immunology, Tokyo, Japan) and VEGF (Immuno-Biological Laboratories, Gunma, Japan) were measured by a sandwich method with enzyme-linked immunosorbent assay kits according to manufacturer’s instructions. The value was represented as per 10^5^ cells.

### 4.6. SCI Model, Intravenous ASC Transplantation, and Functional Evaluation

Spinal cord injury was induced by the weight-drop method [23]. Ten-week-old rats weighing 210–230 g were anesthetized, and dorsal laminectomy was performed at the T10 vertebra. Then, a 10 g weight was dropped onto the spinal cord from a height of 25 mm. Postoperatively, animals were kept warm on highly absorbent bedding, injected with antibiotics, and given manual bladder expression twice daily until reflex bladder emptying returned.

On day eight after SCI, rats were randomly assigned to two groups, and cell transplantation was carried out using the method reported in our previous study [13]. ASCs were incubated in growth medium containing 10 ng/mL basic fibroblast growth factor (bFGF, Thermo Fisher Scientific) for 24 h before transplantation. The cells were detached with trypsin/EDTA and resuspended in physiological saline; 2.5 × 10^6^ cells (in 500 µL) were infused via the tail vein at a rate of 25 µL/min using a stereotaxic microinjector (model 310, Muromachi Kikai, Tokyo, Japan). SCI control animals received the same volume (500 µL) of physiological saline alone.

Hindlimb motor function was evaluated in SCI rats using the BBB score, a locomotor rating scale developed by Basso et al. [24]. A score from 0 (complete paralysis) to 21 (normal gait) was recorded before and after SCI.

### 4.7. Statistical Analysis

Results are expressed as the mean ± standard error (SE). BBB scores are expressed as the median and IQR. Data were analyzed by Dunnett’s test (pairwise comparisons against the control group). Statistical analysis of BBB scores was performed using the Mann-Whitney U-test. The statistical analyses were performed with JMP 13 (SAS Institute Inc., Cary, NC, USA), and *p* < 0.05 was considered statistically significant.

## Figures and Tables

**Table 1:** Efficiency of adipogenic differentiation.

	Fresh	Cryo-2 weeks	Cryo-4 weeks	Cryo-12 weeks
Efficiency	4/4 (100%)	4/4 (100%)	4/4 (100%)	2/4 (50%)

Fresh- or Cryo-ASCs were differentiated into adipocytes (as described in the Materials and Methods section). The number of successful adipocyte differentiation was counted after Oil red O staining, and the ratio was calculated (*n* = 4).

## Abbreviations

ALP	alkaline phosphatase
ASCs	adipose-derived stem/stromal cells
BBB	Basso–Beattie–Bresnahan
bFGF	basic fibroblast growth factor
CT	culture time
Cryo-ASCs	cryopreserved adipose-derived stem/stromal cells
DEX	dexamethasone
DFAT	de-differentiated fat cells
DMEM	Dulbecco’s Modified Eagle’s Medium
DT	doubling time
EDTA	ethylenediamine tetraacetic acid
ES	embryonic stem
FBS	fetal bovine serum
Fresh-ASCs	fresh adipose-derived stem/stromal cells
HGF	hepatocyte growth factor
IBMX	3-isobutyl-1-methylxanthine
iPS	induced pluripotent stem
IQR	interquartile range
MSCs	mesenchymal stem cells
P	passage
PBS	phosphate buffered saline
SCI	spinal cord injury
SE	standard error
VEGF	vascular endothelial growth factor
